# A novel MeCP2 acetylation site regulates interaction with ATRX and HDAC1

**DOI:** 10.18632/genesandcancer.84

**Published:** 2015-09

**Authors:** Somnath Pandey, Glenn E. Simmons, Svitlana Malyarchuk, Tara N. Calhoun, Kevin Pruitt

**Affiliations:** ^1^ Immunology and Molecular Microbiology, Texas Tech University Health Sciences Center, Lubbock, TX, USA; ^2^ Department of Molecular Genetics, University of Texas Southwestern Medical Center, Dallas, TX, USA; ^3^ Molecular and Cellular Physiology, Shreveport, LA, USA; ^4^ LSU Health Sciences Center School of Medicine, Shreveport, LA, USA

**Keywords:** MeCP2, ATRX, HDAC1, epigenetics, SIRT1

## Abstract

Methyl-CpG-binding protein-2 (MeCP2) regulates gene expression by recruiting SWI/SNF DNA helicase/ATPase (ATRX) and Histone Deacetylase-1 (HDAC1) to methylated gene regions and modulates heterochromatin association by interacting with Heterochromatin protein-1. As MeCP2 contributes to tumor suppressor gene silencing and its mutation causes Rett Syndrome, we investigated how novel post-translational-modification contributes to its function. Herein we report that upon pharmacological inhibition of SIRT1 in RKO colon and MCF-7 breast cancer cells, endogenous MeCP2 is acetylated at sites critical for binding to DNA and transcriptional regulators. We created an acetylation mimetic mutation in MeCP2 and found it to possess decreased binding to ATRX and HDAC1. Conditions inducing MeCP2 acetylation do not alter its promoter occupancy at a subset of target genes analyzed, but do cause decreased binding to ATRX and HDAC1. We also report here that a specific inhibitor of SIRT1, IV, can be used to selectively decrease H3K27me3 repressive marks on a subset of repressed target gene promoters analyzed. Lastly, we show that RKO cells over-expressing MeCP2 mutant show reduced proliferation compared to those over-expressing MeCP2-wildtype. Our study demonstrates the importance of acetylated lysine residues and suggests their key role in regulating MeCP2 function and its ability to bind transcriptional regulators.

## INTRODUCTION

Epigenetic marks such as DNA methylation and histone acetylation influence higher order chromatin structure, self-renewal capacity, and cell differentiation programs in a manner that contribute to normal epithelial cell identity [1]. Many of the proteins that interpret these epigenetic marks become aberrantly regulated during tumor progression. Much work has been done to demonstrate the role of epigenetics in regulating cellular processes [2, 3]. Methyl CpG-binding protein 2 (MeCP2) represents one of the central readers of the epigenome in both normal and pathophysiological contexts [4-6]. Since its discovery more than two decades ago, MeCP2 has been studied largely in neuronal systems. Mutation in the MeCP2 gene causes Rett syndrome (RTT), one of the most frequent causes of X-linked neurologic disorders [7]. Prior to this discovery, MeCP2 had been demonstrated to selectively bind CpG dinucleotides in the mammalian genome and assist in transcriptional repression together with histone deacetylases (such as HDAC1) and corepressor proteins [8-11]. These studies established a link between DNA methylation and the deacetylation of chromatin. Apart from MeCP2, other chromosomal proteins, like HP1 and ATRX, are also known to be involved in epigenetic gene silencing [12-14]. Studies have shown that HP1 and ATRX interact with MeCP2 in order to maintain a repressive state of genes. However the nature of such interactions remains incompletely studied. The role of epigenetic regulators traditionally associated with gene repression, such as HDACs, has become more complex in recent years, as more groups demonstrate that the site of action can be both proximal and distal to the effected gene targets. SIRT1, for example, is a member of the +NAD-dependent class III histone deacetylases (HDACs) that regulate broad and complex physiological processes. SIRT1/2 have been shown to negatively regulate the expression of several classic Wnt target genes such as *E-cadherin* and *Secreted Frizzled-Related Protein 1* (SFRP1) [15]. A recent report also has shown how SIRT1 plays a key role in E-cadherin gene silencing and epithelial-mesenchymal transition [16]. However, SIRT1 has also been shown to positively regulate the expression of CYP19A1 gene which encodes for aromatase protein [17], which is frequently increased in estrogen-dependent breast tumors. This finding connected SIRT1 with the positive regulation of gene expression and uncovered an unexpected role. Thus, SIRT1 contributes both to activating growth promoting genes, like aromatase [17] and Wnt pathway activators [18], as well as to the silencing of tumor suppressor genes (TSGs), such as *E-cadherin* and *SFRP1* [15]. This complexity in the regulation of gene expression may explain why SIRT1 has been reported to regulate a wide array of cellular processes, from promoting chemoresistance to conventional chemotherapeutic agents to regulating cell migration [19-21]. The findings reported here which connect SIRT1 and MeCP2 might help explain some of the complexity.

SIRT1 regulates multiple signaling networks that influence cellular responses to stress. A recent study showed that p300 acetyltransferase targets MeCP2 for acetylation and that SIRT1 deacetylase regulates the acetylation of ectopically expressed MeCP2 [22]. However, whether inhibition of SIRT1 was effective in modulating the acetylation of endogenous MeCP2 or its interaction with co-repressor molecules has yet to be adequately addressed. Here we show that inhibition of SIRT1 leads to acetylation of endogenous MeCP2 in multiple cancer cell lines. Additionally, upon selective pharmacological inhibition of SIRT1 in colon and breast cancer cell lines, we identify novel acetyl-lysine residues in endogenous MeCP2, at sites known to be relevant for DNA or protein-protein interaction. We also demonstrate that induced acetylation of MeCP2 did not change promoter binding but decreased MeCP2 binding to ATRX and HDAC1. Our investigation revealed that a small molecular inhibitor of SIRT1 can be used to selectively decrease H3K27me3 repressive marks on silenced gene promoters. Finally we report that RKO cells over-expressing the acetylation mimetic MeCP2-K171Q (K171Q) displayed reduced cell proliferation as compared to those over-expressing MeCP2-wild type (WT). These data presented herein illustrate the regulatory role of SIRT1 upon MeCP2, and how SIRT1 potentiates MeCP2 activity in colon and breast cancer cells.

## RESULTS

### SIRT1 binds and deactylates MeCP2 in multiple cell lines

**Figure 1:**
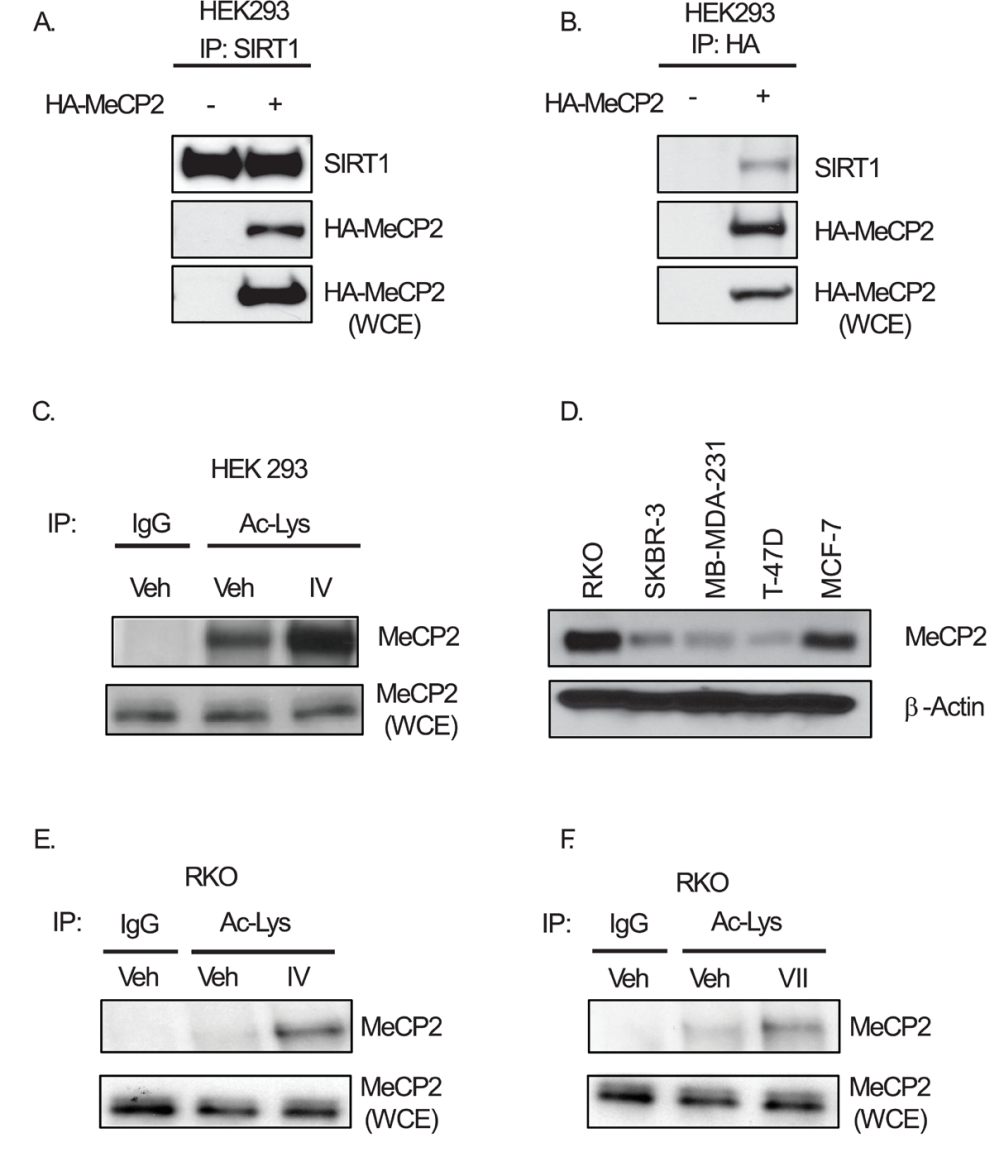
SIRT1 binds to MeCP2 and deacetylates it in multiple cell lines HEK293 cells were transiently transfected with either empty vector or plasmid containing HA-tagged-MeCP2. The protein lysate was extracted and co-immunoprecipiptation was performed using antibodies against either (A) SIRT1 or (B) HA epitope, and the respective co-precipitation of MeCP2 and SIRT1 proteins was assessed using Western blot analysis. (C) HEK293 cells were treated with either vehicle or 20 μM inhibitor IV for 30 minutes and the cells were harvested lysis buffer. Immunoprecipitation was performed using 1.5 μg of either normal IgG or anti-acetyl-lysine antibody followed by Western blot analysis for MeCP2. To ensure inhibitor treatment did not change total levels of MeCP2 whole cell extracts (WCE) were included to analyze total MeCP2 protein levels. (D) Protein was extracted from each of the indicated cell lines and Western blots for MeCP2 and actin was performed on equal total protein. RKO cells were treated with either vehicle or 20 μM inhibitor IV for 30 minutes and the cells were harvested with IP lysis buffer. Immunoprecipitation was performed using either normal IgG or anti-acetyl-lysine antibody followed by Western blot analysis for MeCP2 in RKO treated with either of the SIRT1 inhibitor IV (E), or SIRT1/2 inhibitor VII (F).

We wanted to identify novel deacetylation targets of SIRT1, and previous reports [15] provided clues that MeCP2 could be a critical target. The first step in doing this was finding novel binding partners. We transiently transfected HEK293 cells with either empty vector or HA-tagged-MeCP2 and performed immunoprecipitation using SIRT1-antibody. We found that MeCP2 co-precipitated with SIRT1 (Figure 1A) and this observation was further corroborated by immunoprecipitation of HA-tagged-MeCP2 and blotting for SIRT1 (Figure 1B). We then reasoned that since MeCP2 was binding to SIRT1, it may also serve as an enzymatic target for SIRT1. However, acetylation of endogenous MeCP2 has not been previously demonstrated. Therefore, in order to determine whether endogenous MeCP2 acetylation could be detected upon inhibition of sirtuin deacetylase, we treated HEK293 cells with SIRT1-specific inhibitor IV (IV), an S-35 analog of EX-527 [23] and performed immunoprecipitation with acetyl-lysine antibody followed by immunoblot for MeCP2. We found that SIRT1-specific inhibition indeed caused an increased acetylation of endogenous MeCP2 (Figure 1C). Since MeCP2 is implicated in cancer, we analyzed the relative expression of MeCP2 in our panel of cancer cell lines, by performing western blot analysis of MeCP2 and found that RKO colon cancer and MCF7 breast cancer cells expressed significantly higher protein levels than other cells tested (Figure 1D). We then asked whether SIRT1 mediated deacetylation of endogenous MeCP2 could be detected in RKO cells and indeed, we observed that SIRT1/2 inhibition induced MeCP2 acetylation (Figure 1E and 1F). In MCF-7 cells we observed a similar trend when cells were treated for 2 hours with the same inhibitors (data not shown). Together, these data clearly demonstrate that SIRT1 regulates MeCP2 acetylation levels and pharmacologic inhibition of SIRT1 results in a significant increase in the acetylation of MeCP2 protein without altering total MeCP2 protein levels.

### Endogenous MeCP2 is acetylated at key lysine residues

**Figure 2:**
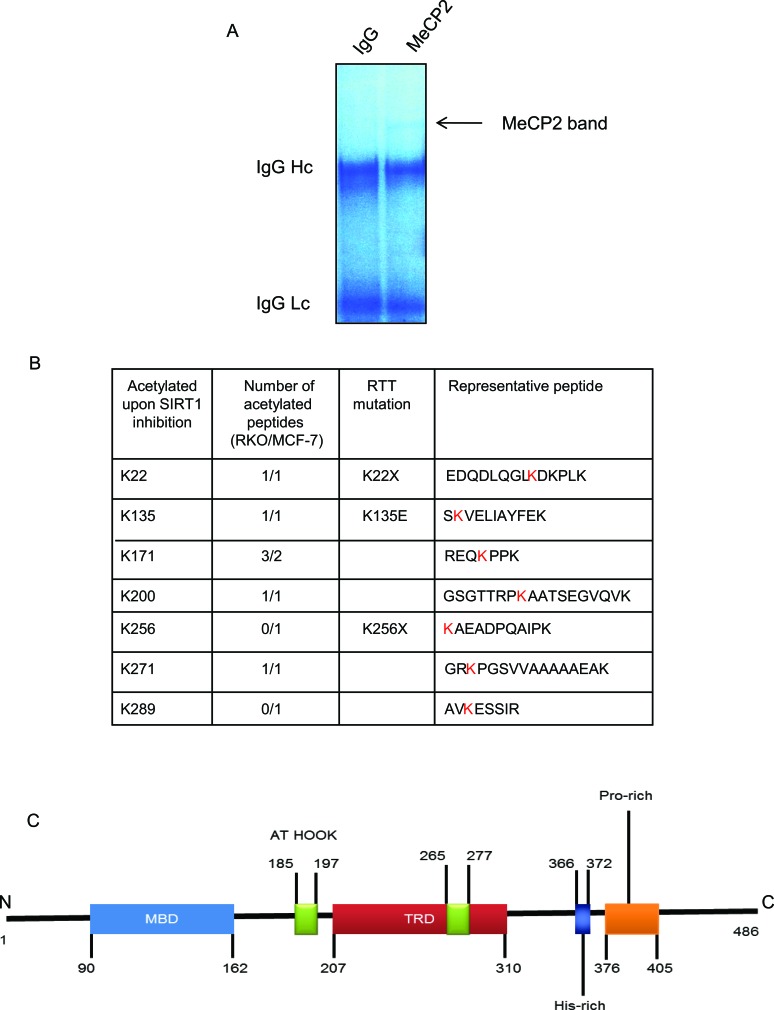
Endogenous MeCP2 is acetylated at key lysine residues (A) RKO and/or MCF-7 cells were treated with DMSO (vehicle control) or 20 μM inhibitor IV and the cells were harvested with IP lysis buffer. Immunoprecipitation was performed for MeCP2 (Sigma, M9317) or specie-matched IgG control and SDS-PAGE was carried out. The gel was stained using Gelcode Blue stain (Thermo Scientific). The gel band corresponding to MeCP2 was excised for analysis by tandem mass spectrometry (MS/MS) for presence of acetylated peptides. (B) Indicated are the putative lysine residues that were found to be acetylated upon SIRT1 inhibition and showed ion peaks at mass/charge (m/z) ratio of ∼126 in RKO and MCF-7 cells. (C) Approximate representation of MeCP2 domain map is shown. N, N-terminal; MBD, Methyl-binding-domain: A-T Hook domain: TRD, Transcriptional repression domain: His-rich, Histidine-rich domain: Pro-rich, Proline-rich domain, C, C-terminal.

In order to further understand the role of acetylation in MeCP2 function, we began to systematically identify the specific lysine residues in endogenous MeCP2 that were acetylated upon SIRT1 inhibition. We inhibited SIRT1 using inhibitor IV in RKO or MCF-7 cells and performed immunoprecipitation for MeCP2. A series of SDS-PAGE gel bands corresponding to MeCP2 such as the one shown in (Figure 2A) were excised for analysis by tandem mass spectrometry (MS/MS). Figure 2B summarizes the putative lysine residues that were found to be acetylated and exhibited ion peaks at mass/charge (m/z) ratio of ∼126 in RKO and MCF-7 cells. We found acetylated lysine residues across the length of the protein, i.e., at the N-terminus, in the methyl-binding domain (MBD), in the intermediate domain (ID) and the transcriptional repression domain (TRD). Some of the identified lysine residues correspond to known RTT mutations. Interestingly, some of the lysine residues detected as acetylation sites (K135, K256 and K271), have been previously reported as sites needed for ubiquitination in SH-SY5Y cells upon ectopic expression of MeCP2 [24].

### K171 is acetylated and mutation affects interaction of MeCP2 with ATRX and HDAC1

**Figure 3:**
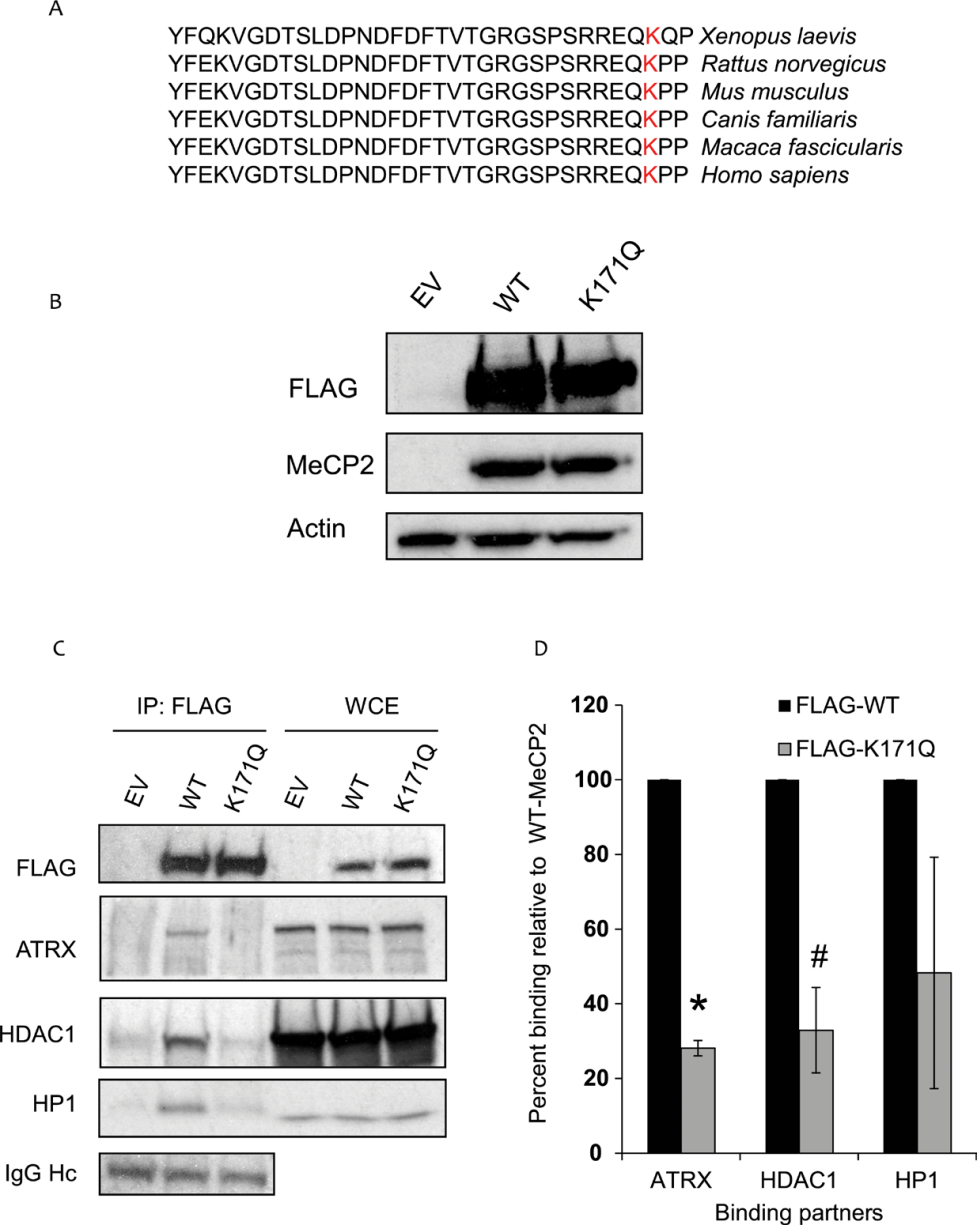
K171 acetylation affects MeCP2′s interaction with its binding partners (A) Alignment of MeCP2 homologs from *Xenopus laevis* to *Homo sapiens*, shows presence of conserved K171 residue. (B) RKO cells were transiently transfected with either empty vector or FLAG-tagged-MeCP2-wild type (WT) or FLAG-tagged-K171Q MeCP2 mutant. Protein lysates were extracted after 24 hours followed by Western blot analysis for FLAG (Sigma) and actin. The FLAG blot was stripped and re-probed for MeCP2 (Sigma, #M9317). A faint band for endogenous MeCP2 is obtained upon over-exposing the X-ray film for ECL detection (data not shown). (C) RKO cells were transiently transfected with either empty vector or a plasmid containing FLAG-tagged-MeCP2-WT or FLAG-tagged-K171Q mutant. After about 24 hours, the protein lysate was extracted and immunoprecipitation was performed using FLAG antibody and the co-precipitation of ATRX/HDAC1/HP1 proteins was assessed. MeCP2-WT shows enriched interaction with the binding partners. K171Q mutant shows decreased binding to ATRX and HDAC1 in a statistically significant manner as indicated by the densitometric analysis. (D) Percent binding relative to MeCP2 was calculated by first taking a ratio of the immunoprecipitated binding partner with K171Q mutant to that of the immunoprecipitated binding partner with MeCP2-WT and then multiplying by 100. NIH ImageJ was used for densitometry. Values are mean ± Stdev, *n* = 3 (ttest results: * represents *p* = 0.002 and # represents *p* = 0.004).

Since acetylated peptides that were identified with lysine 171 (K171) acetylation were more abundant in our mass spectrometry analyses, we focused our attention on the K171 residue exclusively for the remainder of the study. K171 is situated in the relatively uncharacterized intermediate domain (ID), between the methyl-binding domain (MBD) and the transcriptional repression domain (TRD) of MeCP2. Of note, the lysine at this position is conserved in MeCP2 orthologs from humans to Xenopus, suggesting that this residue may be critical to some evolutionarily conserved function of MeCP2 (Figure 3A). Based on this observation, we sought to determine how modulation of K171 acetylation affects MeCP2 function. To determine whether there are functional consequences of acetylating MeCP2 on K171, we mutated K171 to glutamine (K171Q), which mimics constitutive acetylation. Following the construction of the plasmid, we confirmed that MeCP2-WT and K171Q mutant were expressed at similar levels (Figure 3B). Previous studies have shown that MeCP2 interacts with a number of proteins involved in chromatin formation or remodeling, including the histone deacetylase enzyme, HDAC1 [10, 11], heterochromatin protein 1 (HP1α) and ATRX, a SWI2/SNF2 DNA helicase/ATPase that is mutated in ATRX syndrome (α-thalassemia/mental retardation, X-linked) [14]. To determine the role of MeCP2 acetylation in these interactions, MeCP2-WT or K171Q mutant were immunoprecipitated using a FLAG antibody, and the co-precipitation of aforementioned interacting proteins was assessed. Consistently ATRX, HP1α, and HDAC1 were co-precipitated with MeCP2-WT (Figure 3C). However, the K171Q MeCP2 mutant showed a statistically significant decrease in binding to ATRX and HDAC1 (Figure 3D). This suggests that lysine171 is important for MeCP2 interaction with at least two other chromatin remodeling enzymes and that acetylation may serve as a regulatory switch that could potentially modulate protein-protein interaction.

### Pharmacological inhibition of SIRT1 decreases MeCP2′s interaction with ATRX and HDAC1

**Figure 4:**
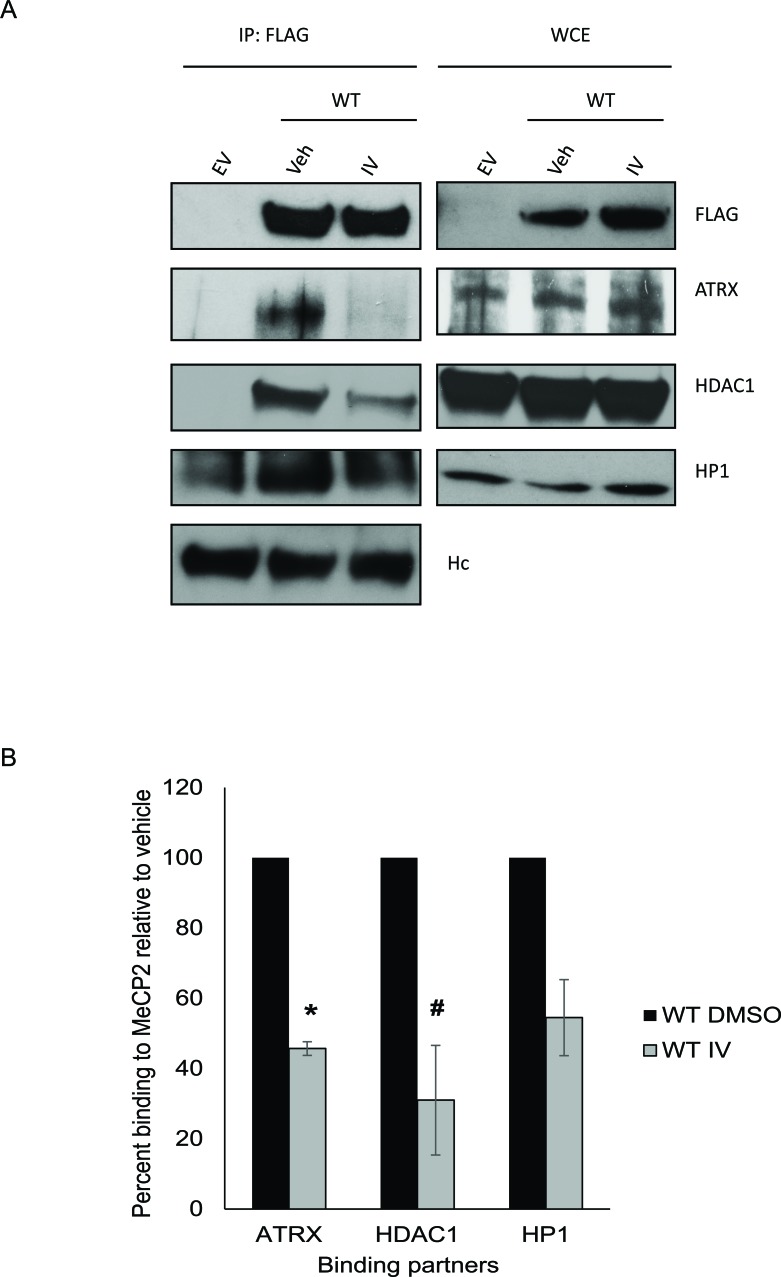
Pharmacological inhibition of SIRT1 decreases MeCP2′s interaction with ATRX and HDAC1 (A) RKO cells were transiently transfected with either empty vector or plasmid containing FLAG-tagged-MeCP2-WT. After about 24 hours, cells were treated with either vehicle or 20 μM inhibitor IV for 30 minutes. The protein lysate was extracted and immunoprecipitation was performed using FLAG antibody and the co-precipitation of ATRX/HDAC1/HP1 proteins was assessed. (B) Pharmacological inhibition of SIRT1 resulted in decreased binding of MeCP2-WT to ATRX and HDAC1 in a statistically significant manner as indicated by the densitometric analysis. Percent binding relative to MeCP2 was calculated by first taking a ratio of the immunoprecipitated binding partner with inhibitor IV treated MeCP2 to that of the immunoprecipitated binding partner with vehicle treated MeCP2 and then multiplying by 100. NIH ImageJ was used for densitometry. Values are mean ± Stdev, *n* = 3 (ttest results: * represents *p* = 0.002 and # represents *p* = 0.013).

To more rigorously address the question of whether MeCP2 interaction with ATRX and HDAC1 is decreased upon MeCP2 acetylation, we treated RKO cells over-expressing MeCP2-WT with a small molecule inhibitor against SIRT1, EX527, previously used by other groups [35], and looked for changes in protein associations. Similarly as with our studies with the K171Q-MeCP2, we noticed a significant decrease in co-precipitation of ATRX and HDAC1 proteins when SIRT1 was inhibited (Supplementary Figure 1). Additionally, treatment with sirtuin inhibitor IV (which we used to identify the acetylated lysines in MeCP2) showed a decrease in the co-precipitation of MeCP2 with the associated proteins to be statistically significant for two of the three proteins studied (Figure 4A and 4B). These data further support the idea that MeCP2 acetylation may be important in modulating MeCP2 protein-protein interactions with ATRX and HDAC1. Here we demonstrate the potential of SIRT1 as a drug target, as SIRT1 inhibition has a direct effect on other epigenetic modifying proteins.

### MeCP2 acetylation induced by sirtuin inhibition does not significantly alter its promoter occupancy at target genes analyzed

**Figure 5:**
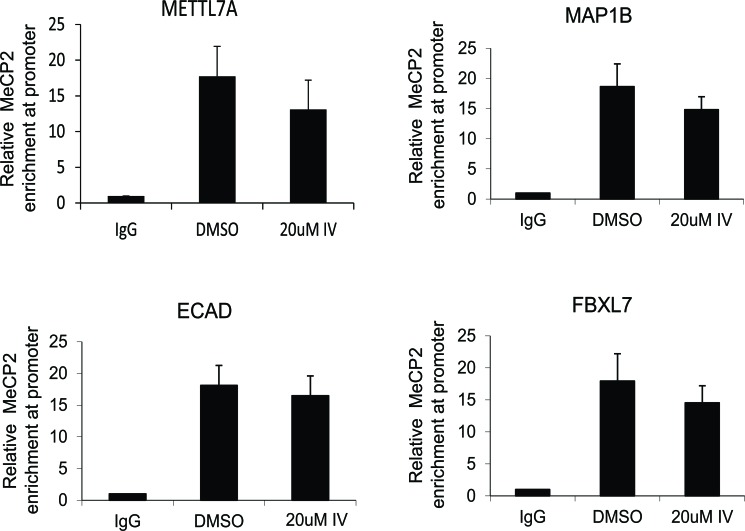
MeCP2 acetylation does not significantly change its promoter occupancy in selected target genes RKO cells were treated with DMSO (vehicle control) or 20 μM inhibitor IV for 2 hours. Equal amounts of purified chromatin DNA from RKO cells was used for ChIP analysis. Antibodies against normal IgG or MeCP2 were used for ChIP analyses, and purified genomic DNA was subjected real-time qPCR analyses using promoter specific primers. ChIP data analysis was performed using fold enrichment method (2-[delta]Ct). At least 3 independent experiments for each treatment were used for each graph. Standard errors are presented.

Next, we wanted to determine whether the pharmacological inhibition of SIRT1 altered MeCP2 occupancy at target genes. Because MeCP2 is known to bind to gene promoters, we became interested in testing whether acetylation altered MeCP2 binding to DNA. Although the E-cadherin promoter had been reported previously to be bound and regulated by MeCP2 [25, 26] and SIRT1 [15], few other genes were known to be co-regulated by both SIRT1 and MeCP2. We sought to identify novel genes co-regulated by SIRT1 and MeCP2, as this class of genes could lead to deeper understanding of how cellular stress influences epigenetic regulation. We screened a number of genes based on reports that they were either MeCP2 targets, or because we identified them in our own RT-PCR screens of SIRT1-depleted cells. Based on these criteria we chose four genes for further analysis including E-Cadherin *(CDH1)*, F-box and leucine-rich repeat protein 7 *(FBXL7),* methyltransferase like 7A *(METTL7A)*, and microtubule-associated protein 1B *(MAP1B)*. Previous study from our lab shows that Aza-cytidine treatment of RKO colon cancer cells resulted in re-expression of ECAD gene [15], indicating that it is basally repressed. To determine the DNA methylation status of the remaining genes, we analyzed gene expression in cells treated with DNA methylation inhibitor, 5-Azacytidine. All of the genes re-expressed upon inhibition of methyltransferase activity, with exception of FBXL7, which was basally expressed (Supplementary Figure 2A). We performed Chromatin-immunoprecipitation (ChIP)-coupled-qPCR analysis, to more rigorously address the question of whether MeCP2 was capable of localizing along the 3kb stretch of the promoters of these genes and whether the dose and duration of inhibitors that induced MeCP2 acetylation could alter its binding at these promoters. ChIP-qPCR analysis revealed enriched MeCP2 binding at several regions along a 3kb stretch of the promoters of all four genes including *CDH1*, *FBXL7*, *METTL7A*, and *MAP1B* (Figure 5). Also, we did not observe any significant enrichment of MeCP2 binding partner, ATRX, at those gene loci (data not shown). Interestingly, while treatment of RKO cells for 2 hours induced acetylation of endogenous MeCP2 (Figure 1, panel E), we did not detect a statistically significant change in MeCP2 occupancy at any of the four genes analyzed (Figure 5). This suggests that the induction of MeCP2 acetylation within a 2 hour window of inhibition that results from pharmacological inhibition of SIRT1 does not alter MeCP2 binding to this subset of gene promoters.

### Pharmacological inhibition of SIRT1 decreases H3K27me3 mark at ECAD, MAP1B and METTL7A gene promoters

**Figure 6:**
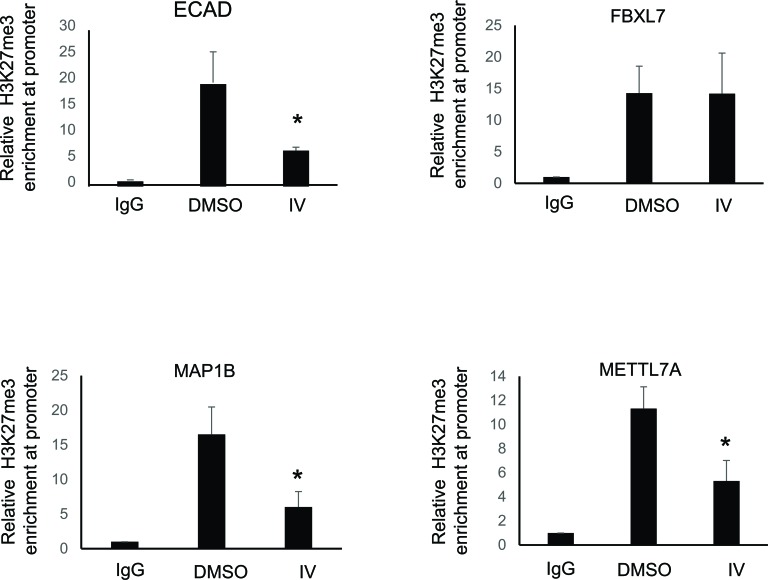
Pharmacological inhibition of SIRT1 decreases H3K27me3 mark at ECAD, MAP1B and METTL7A gene promoter RKO cells were treated with either DMSO (vehicle control) or 20 μM inhibitor IV for 25 hours. Equal amounts of purified chromatin DNA from RKO cells was used for chromatin immunoprecipitation (ChIP). Antibodies against normal IgG or H3K27me3 were used for ChIP analyses, and purified genomic DNA was subjected real-time qPCR analyses using promoter specific primers. ChIP data analysis was performed using fold enrichment method (2-[delta]Ct). Data is representative of 3 independent experiments± SE (ttest results: * represents *p* < 0.05).

Our investigation revealed that MeCP2 is present at the above mentioned regions of repressed gene promoters. Previous studies have demonstrated a significant correlation between DNA methylation status of genes and the histone-PTMs [27]. Therefore, we sought to determine whether a histone modification mark that is known to be stable and strongly correlated with gene silencing, such as H3K27me3 [28, 29], or one which is correlated to transcription initiation, such as H3K4me3 [27], are altered upon SIRT1 pharmacological inhibition. Chromatin immunoprecipitation of H3K27me3 showed enrichment of the H3K27me3 mark at regions along a 3kb stretch of the target gene promoters where MeCP2 was also found to be localized. SIRT1 inhibition resulted in a statistically significant decrease in this repressive mark only at *ECAD, MAP1B* and *METTL7A* gene promoters analyzed (Figure 6). However, we did not see any change in the H3K4me3 histone mark upon SIRT1 inhibition (Supplementary Figure 3).

We have previously demonstrated that selective siRNA mediated knockdown of SIRT1 results in re-expression of E-Cadherin, TSG in RKO colon cancer cells [15]. We reasoned that pharmacologically inhibiting SIRT1 with inhibitor IV, which was used for identifying acetylated sites on endogenous MeCP2, would also alter E-cadherin expression. We treated RKO cells with either vehicle or inhibitor IV and performed semi-quantitative RT-PCR analysis. We found that while E-Cadherin was not expressed basally in RKO cells, inhibitor IV caused its re-expression (Supplementary Figure 2B). We then investigated whether SIRT1 inhibition could alter the expression of the other genes of interest. However, we did not find any significant change in the expression profile of MAP1B and METTL7A genes in RKO cells (data not shown). This might suggest the role of multiple protein factors in regulating gene expression.

### Over-expression of K171Q mutant decreases the proliferation of RKO colon cancer cells

**Figure 7:**
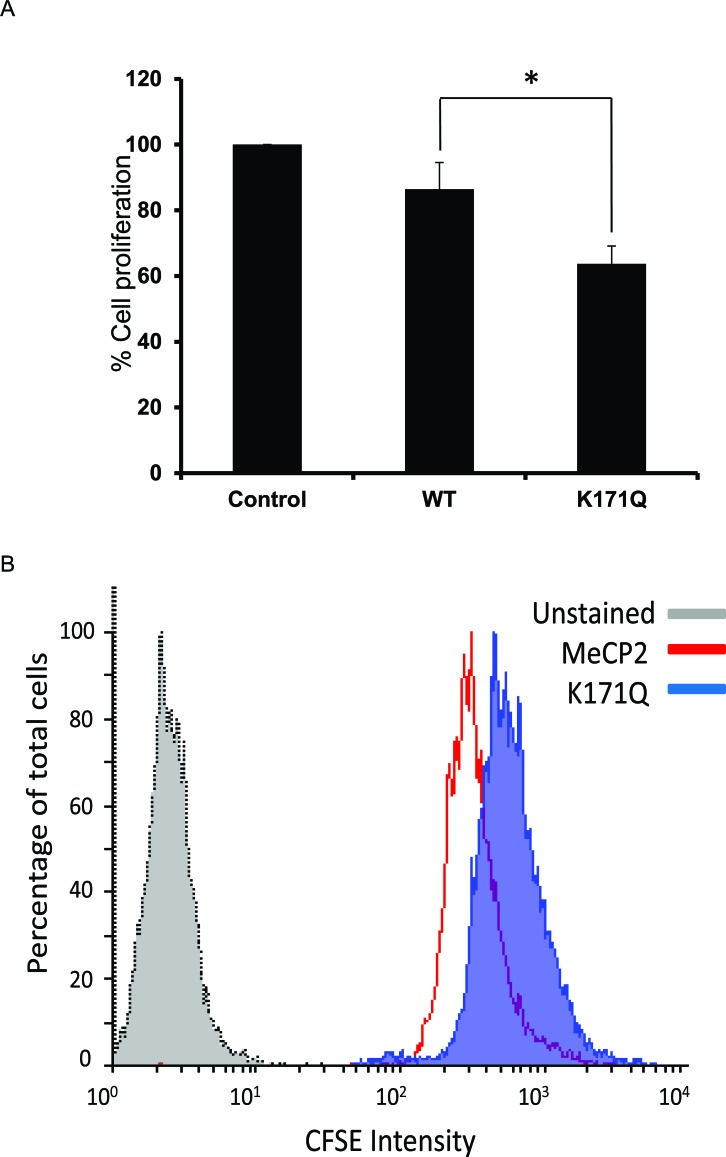
Over-expression of K171Q mutant decreases proliferation of RKO colon cancer cells (A) Stably transfected RKO cells were plated in black 96-well microtiter plate for 72 hours. CellTiter-Blue reagent was added to each well and then incubated for 2 hours at 37°C. Relative fluorescence was quantitated using BioTek Synergy HT plate reader. Values are mean ± Stdev, *n* = 4 (ttest results: * represents *p* = 0.0003). (B) RKO cells were transfected with either WT or K171Q mutant MECP2 plasmid for 24 hours. Following transfection, cells were stained with CFSE as described in methods section. The fluorescent signal of total cell population, excluding dead cell or cellular debris, was measured using FACS Calibur and analyzed using Flowing Software 2.5.1 (Turku Centre for Biotechnology, University of Turku). Figure is a representative of three independent experiments.

Previous works have demonstrated that over-expression of MeCP2 in RKO cells does not induce cell death [30]. Since we had observed such a dramatic difference in protein-protein interactions of MECP2-WT and K171Q mutant, we were interested in the physiologic consequences of over-expressing either the WT or K171Q mutant. To test this, we decided to look at cell metabolism and growth using mitochondrial function as a readout for cell number and viability. Utilizing the Cell Titer-Blue Viability assay, we analyzed cells that were stably expressing either the empty vector, MeCP2-WT or K171Q mutant. We found that the K171Q mutant was associated with retarded cell growth relative to MeCP2-WT over-expressing cells (Figure 7A). To further understand the mechanisms behind how the K171Q mutant affects cell biology, we also analyzed cell proliferation using flow cytometry. We transiently transfected RKO cells with either the WT or mutant constructs then analyzed equal expression of MeCP2-WT and K171Q mutant proteins by Western blot analysis (data not shown). From parallel transfections, we analyzed cell proliferation utilizing a fluorescent cell membrane dye. With this technique, we were able to measure the ability of cells to undergo division over a given period of time. However, unlike the Cell Titer-Blue experiments, we were able to clearly exclude non-viable cells from our analysis. Still, consistent with the results from Cell Titer-Blue assay, we found that cells transiently over-expressing K171Q mutant were dividing at a slower rate than the WT-over-expressing cells (Figure 7B). These data illustrate the importance of SIRT1-MeCP2 activity in the regulation of potent regulatory pathways that ultimately control cell survival and growth signals.

## DISCUSSION

The present study clarifies the link between SIRT1 activity, epigenetic regulation of specific tumor suppressor genes and acetylation of MeCP2. The data also implicate SIRT1-mediated deacetylation of MeCP2 as an important determinant for protein complexes MeCP2 may engage. Furthermore, because cells expressing a MeCP2-acetylation-mimetic mutant showed altered cell proliferation, this further links MeCP2 acetylation with growth control. Based on these findings and others demonstrating that SIRT1 deacetylates specific transcription factors in a context-dependent manner, it is reasonable to conclude that aberrant protein acetylation patterns could potentially alter the expression of critical growth control genes if SIRT1 expression increases beyond a critical threshold [17, 31]. Proteins undergo various post-translational modifications (PTMs) [32, 33], which in turn regulate multiple aspects of their function. The importance of protein acetylation is becoming increasingly evident [34]. For example, reversible lysine acetylation has been shown to regulate the levels, catalytic activity and substrate accessibility of several metabolic enzymes [35]. Lysine acetylation of some transcription factors, such as ERRα, has been shown to decrease its DNA binding [36], while acetylation of p53 has been linked with increased transcriptional activity [31, 37]. Lysine acetylation has also been reported to regulate protein dimerization. For example, cytokine treatment has been shown to stimulate acetylation of Lys 685 on Stat3, enabling formation of stable dimers, a prerequisite for cytokine-stimulated DNA binding and transcription [38]. Thus, depending on the protein and specific lysine residues that are acetylated, this post-translational modification can influence many cellular processes.

Early studies demonstrated that SIRT1 is involved in epigenetic silencing of genes whose promoters are hypermethylated in cancer cells [15]. A more recent study demonstrated that SIRT1 binds and regulates the acetylation of ectopically expressed MeCP2 and is enriched at BDNF promoter in SIRT1 deficient mice [22]. Our current results indicate that the role of increased levels of SIRT1, which is frequently observed in cancer, could contribute to an imbalance in the acetylation of critical lysine residues in MeCP2. While our data demonstrates that SIRT1 inhibition causes an induction of acetylation during the 30 minutes to 2 hour treatment, MeCP2 does not leave the genomic loci of any of our panel of four genes in colon cancer cells. Because the number of promoters that were analyzed was relatively small, the possibility remains that MeCP2 acetylation may also alter its binding to other regions in the genome with CpG methylation. Considering these data, we conclude that MeCP2 may act as a “bridge” between DNA and corepressors in a context-dependent manner.

Histones-PTMs regulate various cellular processes either by recruiting other regulatory molecules or by introducing a change in the chromatin structure mediated by loosening the DNA-histone interaction. Previous studies have demonstrated in colon cancer and breast cancer cells that SIRT1 localizes at the promoter of *ECAD* gene and its long-term inhibition via shRNA results in increased histone H4K16-and H3K9-acetylation respectively, which strongly associates with *ECAD,* TSG re-expression [15]. To our knowledge we provide the first report herein that small molecular inhibitor (drug IV) against SIRT1 can be used to bring about a significant decrease in histone H3K27me3 repressive mark at the gene loci (*ECAD, MAP1B* and *METTL7A*) where MeCP2 was found to be localized. The epigenetic mark, H3K27me3 is a result of the catalytic activity by histone methyltransferase, EZH2, a component of polycomb repressive complex 2 (PRC2), a key regulator involved in maintenance of stem cell identity, X-chromosomal inactivation and differentiation during embryonic development [39, 40]. Previous reports indicate that SIRT1 interacts with PRC2 [3]. This link suggests that SIRT1 might also regulate histone methylation via by influencing EZH2 activity. It is also worth speculating that SIRT1 may potentially regulate H3K27me3 via acting on JMJD3 histone demethylase which brings about removal of the repressive H3K27me3 mark [41].

The findings described here have provided deeper insight into the role of SIRT1 in epigenetic regulation and also illustrates how MeCP2 is regulated through a posttranslational mechanism. Additionally, recent studies have shown that certain MECP2 mutations (R133C and A140V) affect the interaction with ATRX but not with DNA in neuronal system [42] and that MeCP2 recruits ATRX at a subset of imprinted gene domains [13]. Multiple studies have explored the role of MeCP2 and HDAC1 in regulating gene expression [9-11, 43]. Together, our results indicate that MeCP2-WT exists in a complex together with ATRX, HDAC1 and HP1 and suggest that an acetylation-mimetic of MeCP2 or induced acetylation alters this interaction. However, while the lack of binding of ATRX to K171Q mutant supports the acetylation hypothesis, the alternative hypothesis that mutating the interaction site disrupts the interaction and is independent of acetylation cannot be ruled out. It is therefore reasonable to speculate that MeCP2 acetylation patterns regulated by SIRT1 activity may not only influence MeCP2 function in a cancer context, but could also do so in other contexts wherein the specific lysine residue targeted will play an important role in cell-type specific biology. Future work may identify more factors involved in this SIRT1-MeCP2 regulatory network and through such work; our understanding of the key molecular relationships in cancer may lead to more effective epigenetic therapies.

## MATERIAL AND METHODS

### Cell Culture

Cell lines were obtained from the American Type Culture Collection. RKO and MCF-7 cells were cultured in MEM medium supplemented with 10% fetal bovine serum, 1% penicillin/streptomycin (Invitrogen). Media for MCF-7 cells were supplemented with insulin. For inhibitor studies, inhibitor III, VII and IV (EMD-Millipore 566322, 566327, 566325) were resuspended in DMSO and cells were treated with DMSO as a vehicle control and varying amounts of drug as noted in the figure legends.

### Constructs and Transfections

FLAG-tagged-pCDNA3.1(−) and FLAG-tagged-MeCP2-WT-pCDNA3.1(−) (encoding MeCP2 e2 iso-form) were kind gifts from Dr. Paul Wade. FLAG-tagged-K171Q-MeCP2-pCDNA3.1(−) was generated using outward PCR method. Briefly, 3×10^6^ cells were plated in p100 mm dishes and after 24 hours, either FLAG-tagged-pCDNA3.1(−) (empty vector), or FLAG-tagged-MeCP2-WT-pCDNA3.1(−) or FLAG-tagged-K171Q-pCDNA3.1(−) constructs were transfected using Lipofectamine-2000 (Invitrogen) according to the manufacturer's protocol.

### Western Blots

Antibodies used in Western blots were as follows: HDAC1 (Santa Cruz, sc-7872), MeCP2 (Sigma, M9317; Cell Signaling, #3456), SIRT1 (Delta biolabs, DB083), β-actin (Santa-Cruz, sc-47778), ATRX (Santa Cruz, sc-15408), HP1 (Abcam, ab109028) and FLAG-M2 (Sigma, 1804). Membranes were incubated in 5% milk/TBST with primary antibody overnight at 4°C. This was followed by briefly washing the membranes with TBST and probing with horseradish peroxidase (HRP)-conjugated secondary antibodies in 5% milk/TBST for 1 hour at room temperature. Membranes were then washed with TBST before visualization by enhanced chemiluminescence reagent (Pierce).

### Reverse Transcriptase-PCR (RT-PCR)

Total RNA was isolated using Trizol (Invitrogen) and 2 μg of RNA was used to produce cDNA via M-MLV Reverse Transcriptase (Promega). Equal amount of cDNA from each sample was used for end-point PCR using intron-spanning primers for gene expression analysis (RT-PCR). Specific primers against the 5′-regulatory region for human FBXL7, MAP1B, E-cadherin, and METTL7A are presented in the Table 1.

**Table 1:** Primers used for the study

Gene Expression	Forward Primer	Reverse Primer
FBXL7	TCCACCGAATCTCCCAGGAT	CCTGCAGCCACTGACAGTTA
MAP1 B	AGAATGCTGCCAATGCCTCT	CAGGGTCATTCCCACTCACC
METTL7A	CTGCAGTTTGAGCGCTTTGT	GGATCCAGGACTTGTTGCCA
ECAD	GGAAGTCAGTTCAGACTCCAGCC	AGGCCTTTTGACTGTAATCACACC
β-actin	GGACTTCGAGCAAGAGATGG	AGCACTGTGTTGGCGTACAG
5′ regulatory region (ChIP)		
MAP1B	GCCTGACTAGTGCAACTCCA	GAAGGGGGAAGACGCTCTCA
FBXL7	GCTCTGTGCTTGCCTCACTA	AAATGGCCCTTTGGTGTTCG
METTL7A	AGCACTCTTCTGGTGTGGTT	CCCTTCTCTGGGTTTGGAGAC
ECAD	TACAAAATTAGCCGGTGTGGTG	GATTTCAGGTGTGAGCCATGAG

### Chromatin Immunoprecipitation (ChIP) and Real Time Quantitative PCR

RKO cells were treated with 20 μM of inhibitor IV or DMSO (control) for 2 hours. After the treatment, cells were subjected to 1% formaldehyde cross-linking (Sigma) for 10 minutes at room temperature. The cross-linking reaction was quenched by adding glycine (Sigma) to a final concentration of 0.125M for 5 minutes at room temperature. The medium was then removed and cells were washed twice with cold PBS. Cells were scraped in PBS and pelleted. Pellet was resuspended in SDS Lysis Buffer (Millipore 20-163) and sonicated in a Diagenode Bioruptor sonicator. The soluble chromatin fraction was collected and incubated overnight at 4°C with either MeCP2 (Sigma, M9317), or H3K27me3 (Millipore, 07-449), or H3K4me3 (Cell Signaling, 9751S) or rabbit IgG (Sigma I5006). Protein A magnetic beads (Invitrogen) were added to the chromatin-antibody mixture and incubated with rotation for 2.5 hours at 4°C. Beads were washed with a low salt wash buffer (Millipore 20-154), high salt wash buffer (Millipore 20-155), and TE (Millipore 20-157). Reverse-crosslinking was performed at 65°C overnight followed by treatment with proteinase K (Promega) at 55°C for 2 hours and RNaseA for 2 hours at 37°C. DNA was eluted using Qiaquick PCR purification kit (Qiagen) and amplified as described above. SYBR Green Real-Time PCR was used to quantify the relative enrichment of MeCP2 or H3K27me3 or H3K4me3 along the 3 kb stretch of promoters of target genes. ChIP primer sequences are presented in the above table. ChIP data analysis were performed using fold enrichment method (2-[delta]Ct) and at least 3 independent experiments for each treatment were used for each graph. The Bio-Rad CFX-96 real-Time PCR System and PerfeCTa SYBR green FastMix, ROXTM (Quanta Bioscience, Inc., MD, cat. number 95073) were used for real-time analysis.

### Immunoprecipitation

After a 30′ to 2 hours treatment of RKO or MCF-7 cells, they were harvested either in IP lysis buffer (25mM Tris HCl pH7.5, 150mM NaCl, 1% NP-40, 1mM EDTA, 5% Glycerol, 1x Protease inhibitor cocktail). The total protein concentration was measured in all samples by standard BCA protocol (Thermo Scientific) to ensure that equal amount of protein was added to each sample. IP was performed overnight at 4°C using either 1.5 μg of normal mouse IgG (control) or acetyl-lysine antibody (Cell Signaling, #9681) or anti-MeCP2 antibody (Sigma). The following day immune-complexes were incubated for 2.5 hours at 4°C with Protein A Dynabeads (Invitrogen). Immunoprecipitates were washed in the same buffer, boiled and subjected to sodium dodecyl sulphate polyacrylamide-gel electrophoresis (SDS-PAGE) using Bio-Rad mini system (Bio-Rad). The blots were then processed as mentioned above.

### Mass spectrometry

RKO and/or MCF-7 cells were treated with DMSO (vehicle control) or 20 μM inhibitor IV and the cells were harvested with IP lysis buffer. Protein extract was obtained and total protein concentration was estimated using BCA method (Thermo Scientific). Immunoprecipitation was performed for MeCP2 (Sigma, M9317) or species-matched rabbit IgG control, overnight at 4°C. Protein A Dynabeads (Invitrogen) were added the following day to the immune-complex and incubated again for 2.5 hours at 4°C. Immunoprecipitates were washed four times, denatured and SDS-PAGE was performed. The gel was fixed in fixing solution (methanol-acetic acid solution prepared in milliQ water (40% methanol, 10% acetic acid)) and stained overnight using Gelcode Blue stain (Thermo Scientific). Sample bands corresponding to appropriate molecular weight mark for MeCP2 were excised and shipped to Applied Biomics Inc. (Hayward, CA) for acetylation site identification by mass spectrometry.

### Co-immmunoprecipitation

RKO cells transiently transfected with either empty vector or MeCP2-WT or K171Q MeCP2 mutant were washed with ice-cold PBS and lysed in co-IP lysis buffer (50mM Tris-HCl pH 7.5, 100mM NaCl, 1% NP-40, 10% Glycerol, 1mM DTT, 0.5mM EDTA, 0.5mM EGTA, 0.5mM NaF and Protease inhibitor cocktail). This was followed by quantification for equal protein loading by standard BCA protocol (Thermo Scientific). Cell extracts were incubated with 1.5 μg of FLAG-M2 antibody. Separate lysate tube was prepared from samples for incubation with species-matched normal mouse IgG. Samples were incubated overnight with gentle rotation at 4°C. Protein G Dynabeads (Life Technologies) were incubated with the antigen-antibody complex for 2.5 hours the following day. Beads were washed four times with lysis buffer with gentle agitation for 5 minutes per wash. 2x Laemmli sample buffer (Bio-Rad) was used for elution of complex from beads followed by Western blotting along with whole cell extract.

### Cell-Titer Blue assay

To analyze cellular metabolic function we utilized Cell Titer-Blue Kit from Promega. We plated 1000 RKO cells that had been stably transfected with MECP2-WT or K171Q mutant into black fluorescence compatible 96-well microtiter plates. Cells were allowed to grow in culture for 3 days. On the final day in culture, Cell Titer-Blue reagent was added to each well and incubated for 2 hours at 37°C. Cells were then analyzed for fluorescence at 560/590nm emission using a BioTek plate reader and Gen5 software. The data were quantitated and compiled from four independent experiments.

### CFSE staining

To determine cell proliferation, cells over-expressing either MeCP2-WT or K171Q mutant were stained with carboxyfluorescein succinimidyl ester (CFSE) from Cell Trace Proliferation kit from Invitrogen. Cells were stained according to manufacturer's guidelines. 1×10^6^ cells/ml were incubated in a suspension with 0.1% BSA in PBS. Cells were incubated with 10μM of CFSE for 10 minutes at 37°C. Residual stain was removed from cells and cells were placed back at 37°C for 72 hours. Cells were harvested using 1mM EDTA, and then resuspended in FACS buffer (PBS, 2% FBS, 0.1% sodium azide). Cells were analyzed using FACS Calibur.

## SUPPLEMENTARY MATERIAL FIGURES


