# Supplementary material for: A novel MeCP2 acetylation site regulates interaction with ATRX and HDAC1
Source: Genes Cancer. 2015 Sep;6(9-10):408–21. doi: 10.18632/genesandcancer.84 (PMC4633168; doi:10.18632/genesandcancer.84)
Supplement: Supplementary file 1 [file ganc-06-408-s001.pdf]

A novel MeCP2 acetylation site regulates interaction with ATRX and HDAC1

Supplementary Material

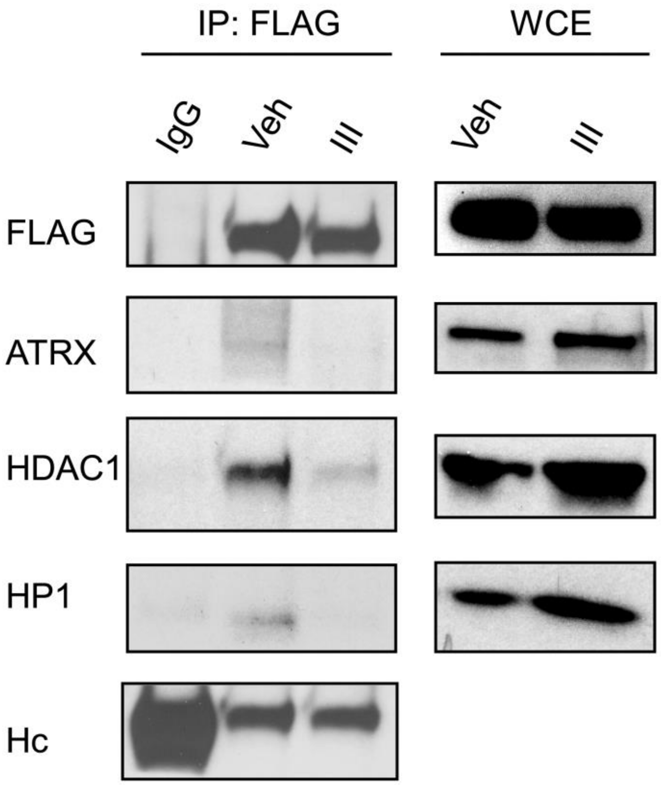

Supplemental Figure 1. Pharmacological inhibition of SIRT1 decreases MeCP2's interaction with ATRX and HDAC1. RKO cells were transiently transfected with either empty vector or plasmid containing FLAG- tagged-MeCP2-WT. After about 24 hours, cells were treated with either vehicle or 50 μM inhibitor III for 30 minutes. The protein lysate was extracted and immunoprecipitation was performed using FLAG antibody and the coprecipitation of ATRX/ HDAC1/ HP1 proteins was assessed.

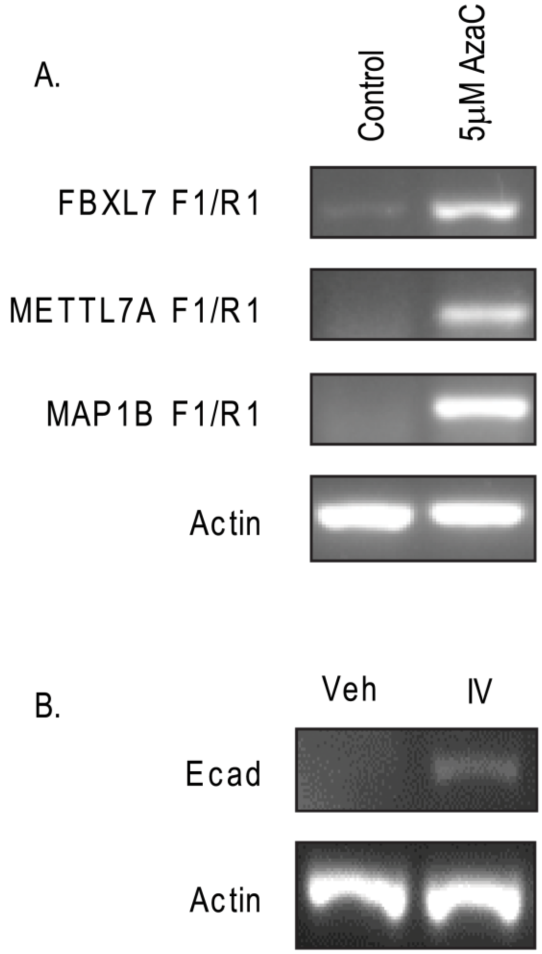

Supplementary Figure 2. Inhibition of DNA methylation or SIRT1 deacetyase results in target gene re-expression. (A) RKO cells were treated with either Control or 5 µM of 5'azacytidine for 48 hours. RNA was isolated and subjected to RT-PCR using Intron-spanning primers. (B) Pharmacological inhibition of SIRT1 results in E-Cadherin re-expression. RKO cells were treated with either vehicle or 20 µM of inhibitor IV for 24 hours. RT-PCR using equal amounts of cDNA from each sample was performed to determine the level of expression of E-Cadherin gene. Data shown are representative of at least 3 independent experiments.

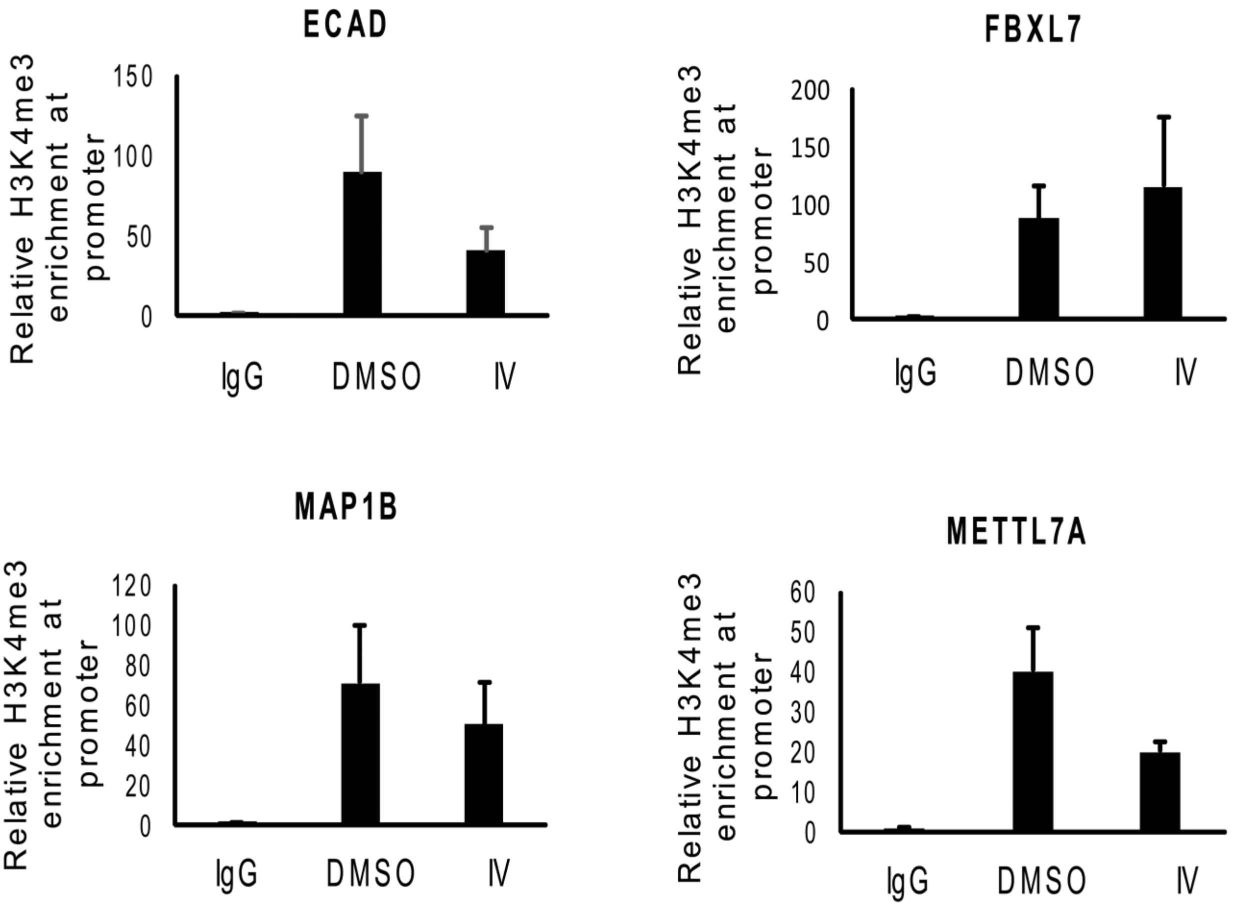

Supplementary Figure 3. Pharmacological inhibition of SIRT1 does not alter H3K4me3 mark at target gene promoters analyzed. RKO cells were treated with DMSO (vehicle control), 20 μM inhibitor IV for 25 hours. Equal amounts of purified chromatin DNA from RKO cells was used for chromatin immunoprecipitation (ChIP). Antibodies against normal IgG or H3K4me3 were used for ChIP analyses, and purified genomic DNA was subjected real-time qPCR analyses using promoter specific primers. ChIP data analysis was performed using fold enrichment method (2-[delta]Ct). Data is representative of 3 independent experiments. Standard errors are presented.
